# Pomegranate Peel as a Source of Bioactive Compounds: A Mini Review on Their Physiological Functions

**DOI:** 10.3389/fnut.2022.887113

**Published:** 2022-06-09

**Authors:** Yaxian Mo, Jiaqi Ma, Wentao Gao, Lei Zhang, Jiangui Li, Jingming Li, Jiachen Zang

**Affiliations:** ^1^College of Food Science and Nutritional Engineering, China Agricultural University, Beijing, China; ^2^College of Forestry and Landscape Architecture, Xinjiang Agricultural University, Ürümqi, China

**Keywords:** pomegranate peel, food by-products, bioactive compounds, physiological functions, resources reuse

## Abstract

The production and consumption of pomegranates have always been increasing owing to their taste and nutrition. However, during fruit processing, a large number of by-products are produced, such as peels and seeds, which can lead to environmental pollution problems if not handled properly. The pomegranate peel takes up about 26–30% of the total weight, while it contains abundant bioactive substances. This paper carries out a mini review of the characterization and physiological functions of key bioactive compounds in pomegranate peel, comprehensively assessing their effects on human health. The overview summarizes the main phenolic substances in pomegranate peel, including tannins, flavonoids, and phenolic acids. Dietary fiber and other bioactive substances such as alkaloids, minerals, and vitamins are also mentioned. These components act as antioxidants by improving oxidative biomarkers and scavenging or neutralizing reactive oxygen species, further contributing to their extensive functions like anti-inflammatory, anti-cancer, antibacterial, and cardiovascular protection. Overall, it is envisaged that through the deeper understanding of bioactive compounds in pomegranate peel, the waste sources can be better reused for physiological applications.

## Introduction

One-third of the food produced for human consumption in the world has been lost or wasted. In quantitative terms, this represents 1.3 billion tons and a cost of about 990 billion dollars, including food processing waste and food losses (1, 2). Among these, the fruit and vegetable processing industry is one of the largest by-products producers, approximately accounting for 45% (3). The generated processing by-products cause not only the waste of resources but also cause environmental pollution problems if not properly treated.

Pomegranate, belonging to the *Punica* L. genus, Punicaceae family, originated in Iran, India, China, and the Mediterranean region in 3000 B.C. (2, 4). Nowadays, it is also cultivated in North and tropical Africa, North and South America, and Caucasus area in addition to the areas mentioned above (5, 6). The production and consumption of pomegranates keep increasing owing to their taste and nutrition. According to statistics, the global production of pomegranate was approximately 3.8 million tons in 2017 (7). The fruit of pomegranate can be divided into three parts, which are peels, juice, and seeds. Usually, pomegranates are consumed fresh or processed into juice. When processed into pomegranate juice, a large amount of waste is generated, in which peels take up about 26–30% of the total weight (8). It is worth noting that pomegranate peels contain many bioactive compounds such as polyphenols, dietary fiber, vitamins, minerals, etc. (9, 10). Numerous *in vitro* and *in vivo* studies have shown that these substances have a broad range of biological activities and health benefits, such as antioxidant, anti-inflammatory, anti-cancer, and so on (11–14). In addition, their presence is associated with the prevention and treatment of several chronic metabolic diseases including cardiovascular diseases, diabetes, and obesity (15, 16). Therefore, the bioactive components in pomegranate peels can be exploited as functional ingredients to better utilize the by-product resources, further providing added value to the pomegranate industry.

This mini-review explores the characterization of key bioactive compounds and physiological functions of pomegranate peels, comprehensively assessing their effects on human health, and discusses potentially future directions for research and practice.

## Bioactive Compounds

**FIGURE 1:**
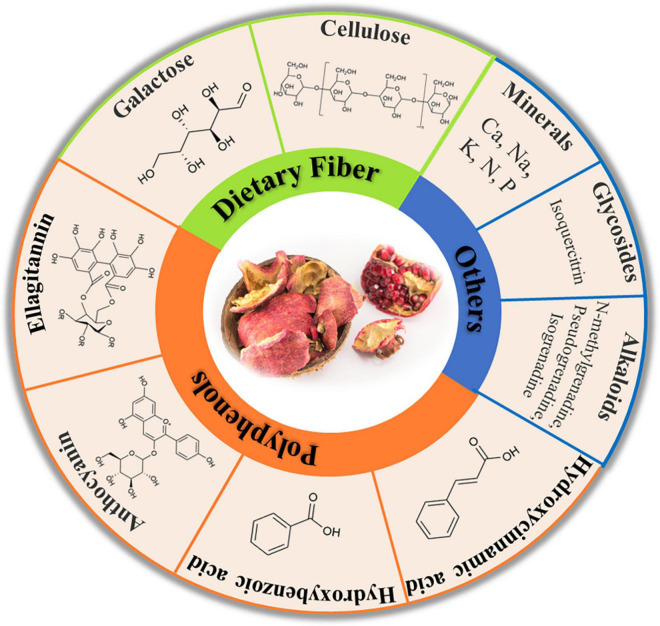
Key bioactive compounds of pomegranate peels.

Since ancient times, pomegranate peels have always been used as folk medicines, owing to their numerous beneficial compounds. In general, the contents of bioactive compounds in peels tend to be higher than in edible parts (17–19). It is also worth noting that complex bioactive compounds in pomegranate peel often exist in the form of a mixture, so the synergistic effect of different compounds can produce a variety of physiological activities (17). The categories and detailed structure of the compounds were drawn in Figure 1.

### Polyphenols

Phenolic compounds are the main secondary metabolites of the shikimic acid, pentose phosphate, and phenylpropanoid pathways and include a large number of water-soluble substances (20). According to the reports, the amount of total phenolics is between 18 and 510 mg/g dry matter in pomegranate peel differed by species, extraction solvents, and extraction methods (21). Their structures are composed of at least one aromatic ring with one or more hydroxyl substituents (22, 23). The main phenolic substances among them are tannins, flavonoids, and phenolic acids (24). Among them, the contents of tannins and flavonoids are between 193 and 420, 84, and 134 mg/g dry matter in pomegranate peel (25).

#### Tannins

Pomegranate peel is rich in several structural forms of tannins, all of which are water-soluble phenolics, and mostly hydrolyzable. Based on their structural characteristics, these compounds can be divided into four main groups, namely, gallotannins, ellagitannins, complex tannins, and condensed tannins (26). As a kind of ellagitannins, punicalagin is the main constituent of pomegranate peel tannins, and the characteristic substance of pomegranate peels, with much higher content than other fractions (16.67–245.47 mg/g dry matter) (27). Punicalagin can produce ellagic acid through spontaneous endo-esterification hydrolysis of the hexahydroxybenzoic acid structure. Then ellagic acid can combine with sugar ligands and polymerize to form ellagitannins with complex structures (28). Their antioxidant effects are provided by polyphenol hydroxyl groups that can reduce the content of free radicals. Besides, the catechol hydroxyl groups in complex and condensed tannins give them the ability to chelate iron and transition metals (29). Tannins may also exert antibacterial activity through the following mechanisms: inhibition of enzyme activity, precipitation of membrane proteins, and depletion of metal ions (30).

#### Flavonoids

Flavonoids mainly refer to a class of compounds derived from flavanone (2-phenylchromanone), which is widely distributed in pomegranate peels. It consists of 15 carbon atoms arranged in the C_6_–C_3_–C_6_ configuration. The structure consists of two aromatic rings (A and B), usually in the form of a heterocyclic ring attached to a third carbon chain (C) (31). The different substitution patterns in the ring form several different subclasses, namely, flavonoids, flavonols, proanthocyanidins, and anthocyanidins.

Rice Evans (32) and Balasundram et al. (33) found that flavonoids have antioxidant activity, which is determined by the structural features and substitution properties as follows: (1) the location and number of hydroxyl groups in A and B rings; (2) the double bond between C_2_ and C_3_, which is conjugated to the 4-keto group on the C ring and enhances free radical scavenging capacity; (3) the presence of double bond between C_2_ and C_3_, partially conjugated to the 3-hydroxyl group on the C ring and enhances free radical scavenging capacity; and (4) hydroxyl groups substituted by glycosylation reduces antioxidant activity. Based on such high antioxidant activity, flavonoids are often called phytoestrogens, which may reduce the incidence of hormone-involved cancers (34, 35).

#### Phenolic Acids

Phenolic acids include gallic, ellagic, caffeic, chlorogenic, butyric, erucic, ferulic, and cinnamic acids, which have been identified in pomegranate peels (36–38). Phenolic acid profiles of pomegranate and their concentrations vary depending on the geographical environment where they are planted. Gallic acid, ellagic acid, and caffeic acid are identified and quantified from six ecotypes of Tunisian pomegranate peels with average contents of 123.79, 35.89, and 20.56 mg/100 g, respectively (38). Phenolic acids consist of a phenolic ring and an organic carboxylic acid (C_6_–C_1_ backbone) and are divided into two groups: hydroxybenzoic acid (C_6_–C_1_) and hydroxycinnamic acid (C_6_–C_3_).

Sanchez-Maldonado (39) studied the antibacterial mechanism of phenolic acids, proving that the transmembrane diffusion of phenolic acids can result in cytoplasmic acidification, and cause cell death in some cases. Interestingly, with more hydroxyl groups, the hydroxybenzoic acid exhibits lower antibacterial activity. The antibacterial activity of hydroxycinnamic acid strongly depends on the double bond of the side chain.

### Dietary Fiber

The dietary fiber is the most abundant component in pomegranate peels, ranging from 33% to 62%, therefore it can be used as a natural source. Hasnaoui et al. (40) compared the content and composition of dietary fiber in the by-products of 12 species of pomegranates. There were lignin, cellulose, uronic acid, and neutral sugars in pomegranate peels after separation. Among them, lignin had the highest concentration. Cellulose and uronic acid had similar contents, only second to lignin, ranging from 16 to 22/100 g (41). Xylose, arabinose, and galactose were mainly constituted in the neutral sugars in pomegranate peels (42). Figure 1 shows the structure of cellulose and galactose. Mari et al. (43) compared the dietary fiber’s composition and physicochemical properties of lemon, grape, pomegranate, and citrus. They found that the ratio of insoluble and soluble dietary fiber in pomegranate was close to 1, which exerted a marked effect on cholesterol absorption area. According to Colantuono’s work (44), the dietary fiber of pomegranate peel was well used in the preparation of alginate microspheres, which showed even higher antioxidant activity than commercial products. In summary, pomegranate peels are valuable sources of dietary fiber with good physicochemical and functional properties.

### Others

In addition to polyphenols and dietary fiber, alkaloids, vitamins, steroids, and various mineral elements are also distributed in pomegranate peels. Neuhofer (45) identified two isomers in pomegranate peels containing acetyl grenadine, 2-(2-hydroxypropyl)-Δ′-piperidine, sedridine, and *N*-acetyl sedridine, belonging to alkaloids. In addition, pseudo grenadine, *N*-methyl grenadine, and iso-grenadine were also discovered in pomegranate peels (46). Besides, pomegranate peels contain many mineral elements such as K, P, Na, Ca, Mg, and N, which have an important role in maintaining the normal physiological functions of the body (9, 10).

## Physiological Functions

### Antioxidant

According to the research of the Institute of Medicine (U.S.), the dietary antioxidant is derived from food, which can significantly reduce the adverse effects of reactive oxygen and nitrogen species on normal physiological functions in humans (47). As previously mentioned, pomegranate peel is a good source of natural antioxidants and the antioxidant capacity of the extracts has been demonstrated in several studies.

Andrés (48) investigated the antioxidant capacity of aqueous extracts from pomegranate and red grape by-products. Pomegranate by-products had a total phenolic content of 134.79 mg gallic acid equivalents (GAEs)/g of extracts, which was higher than that of the red grape by-product extracts (32.16 mg GAE/g of extracts). Thus, it has the potential to become a raw material for pharmaceutical formulations. *In vitro* studies have shown its high antioxidant capacity, mainly attributed to ellagitannins (49). Urolithin, a natural metabolite of ellagitannins, is divided into Urolithin A (Uro-A) and Urolithin B (Uro-B). They exhibit lower antioxidant capacity than the precursors of urolithin (50). Uro-A played a protective effect on cisplatin-induced renal oxidative damage in the mice’s kidneys *in vivo* (51). However, the evidence for urolithin as an antioxidant molecule is weak and more research is needed. Gouda et al. (52), Guo et al. (53), and Rosenblat et al. (54) found that pomegranate peel extracts (PPEs) reduce the levels of thiobarbituric acid reactive substances (TBARS), oxidized low density lipoprotein (Ox-LDL), lipid peroxidation, and oxidative biomarkers associated with cardiovascular risk in healthy subjects, exhibiting potential antioxidant activity.

### Anti-inflammatory

Inflammation is a physiological response to injury that protects the host from invasion by organisms and provides healing to restore homeostasis in the body. As a result of tissue damage, several inflammatory mediators such as chemokines, cytokines, vasoactive amines, eicosanoids, and protein hydrolysis cascade products are synthesized and secreted (55).

In 2008, Shukla et al. (56, 57) demonstrated the anti-inflammatory properties of pomegranate exact by using an animal model of rheumatoid arthritis. Following 13.6 mg/kg of treatment, it was observed that pomegranate extract could lower interleukin-6 (IL-6) and interleukin-1 beta (IL-β), consequently decreasing the arthritis incidence. A preliminary study showed that PPE consumption was effective in reducing clinical symptoms and significantly improving serum oxidative status in patients who had rheumatoid arthritis (58). Similarly, in a randomized controlled trial conducted in patients with rheumatoid arthritis, disease activity was significantly reduced after 8 weeks of PPE supplementation (59). Houston et al. (60) investigated the anti-inflammatory activity of a kind of PPE. The authors verified a marked anti-inflammatory impact of PPE on the expression of cyclooxygenase-2. In obese volunteers, extracts from pomegranate peels exerted anti-inflammatory effects by significantly reducing inflammatory markers [malondialdehyde (MDA), IL-6, and hypersensitive-C reactive protein (hs-CRP)] (61). In 2010, an in *vivo* study on intestinal inflammation found that Uro-A is a potent anti-inflammatory molecule (62). Kamali et al. (63) studied the efficacy of PPE in 62 volunteers with ulcerative colitis. Lichtiger colitis activity index (LCAI) decreased in volunteers after a few weeks of intake. However, there were no significant differences compared to placebo. Therefore, despite the certain evidence for the anti-inflammatory properties of urolithins, more research are needed for application to human practice.

### Anticancer

Cancer causes high mortality rates in both developed and developing countries. Therefore, taking proper preventive measures and early detection is crucial to the treatment of cancer. Punicalagin and ellagic acid have been proved to exhibit significant anticancer activity in a variety of *in vivo* and *in vitro* research. However, it remains an open question whether these molecules play an anticancer role through themselves or their derived microbial metabolites.

Punicalagin and ellagic acid induce apoptosis in prostate cancer cells through some basic pathways, such as the introduction of cytochrome *c* in the cell cytoplasm, upregulation of Bax, and downregulation of B cell lymphoma/leukemia-2 (Bcl-2) (64). Paller et al. (65) studied the impact of PPE on prostate cancer in patients with elevated prostate specific antigen (PSA) levels. The results demonstrated that the ingestion of PPE reduced PSA levels by 13%. In addition, two different clinical studies showed that different conjugated urolithins were identified in the human prostate after pomegranate juice and PPEs depletion (66, 67). This helps to identify molecules accumulated in the human prostate and to explore the role of prostate cancer biomarkers. Similar results were obtained in a recent randomized controlled trial. After 52 weeks of PPE intaking in humans with high-risk prostate cancer, 8-hydroxy-2′-deoxyguanosine (8-OHdG) was reduced in tumor tissue, androgen receptor expression was downregulated in adjacent tissues, and urolithin was also detected in prostate tissue. However, there were no significant effects on insulin-like growth factor-1 (IGF-1), insulin-like growth factor-binding protein 3 (IGFBP-3), free testosterone levels, PSA doubling times, and biopsy indices compared with the placebo group (68).

Estrogen stimulation causes breast cancer cell proliferation and estrogen receptor-positive tumors, which can be examined through anti-aromatase compounds. Uro-B, a metabolite of ellagitannin from pomegranate, is identified as having the most anti-aromatase active component by the aromatase assay and can inhibit testosterone-induced proliferation of Michigan Cancer Foundation-7 (MCF-7) cells (69). Extracts from pomegranate peel have been proved to induce apoptosis of human breast cancer cells (MCF-7). Previous studies have clarified that the combined application of PPE and genistein can inhibit MCF-7 expression in breast cancer cells to a greater extent. Moreover, PPE has the capacity to inhibit the cell proliferation and the expression of angiogenic markers and activate pro-survival signal pathways (70, 71). In addition, Koyama et al. (72) studied the relationship between PG-induced apoptotic system LAPC4 and insulin-like growth factors (IGFs)/insulin-like growth factor binding protein (IGFBP) in prostate cancer cells. They found that pomegranate extracts and IGFBP-3 showed a similar effect of stimulating apoptosis by inhibiting the increase of c-Jun N-terminal kinase (JNK) phosphorylation caused by cell growth, reducing the activation of protein kinase B (PKB/Akt) and mammalian target of rapamycin (MTOR). In addition, a *in vitro* study has shown that ellagic acid in pomegranate can interfere with T24 bladder cancer cells by inducing cell cycle G0/G1 arrest and reducing the expression of Cdk2 gene (73). In conclusion, the results of these different studies suggest that pomegranate peel has a chemo-preventive effect on prostate cancer, breast cancer, bladder cancer, partly related to ellagic acid and its derived metabolite urolithin.

### Anti-bacterial

Just like the antioxidant capacity of pomegranate peel, the antibacterial activity can attribute to the phenolic compounds, with the potential to prevent or treat infections. This is due to their ability to precipitate membrane proteins and inhibit enzyme activity, thus leading to bacterial death and exerting the antibacterial effects (74–77). Al-Zoreky et al. found that the uptake of PPE effectively inhibited *Listeria monocytogenes*, *Staphylococcus aureus*, *Escherichia coli*, and *Yersinia pestis* in small intestinal colitis (78). A study conducted by Panichayupakaranant et al. (79) demonstrated the antibacterial activity of PPE, which contained 13% (w/w) ellagic acid. They found that 2 mg/disk PPE can inhibit the Gram-positive bacteria *Propionibacterium acnes*, *S. aureus*, and *S. epidermidis*. Owing to the antibacterial capacity, natural plant extracts have been broadly applied in the preparation of packing membranes. Hanani et al. compared the antibacterial effect of extracts from pomegranate peels, papaya peels, and pineapple peels, respectively, finding that only the membranes incorporating PPE inhibited all the tested bacteria, i.e., *L. monocytogenes*, *Bacillus cereus*, *E. coli*, and *Salmonella* (80).

### Cardiovascular Protection

Due to irrational lifestyle, some metabolic syndrome diseases such as hypertension, hyperlipidemia, obesity, glucose intolerance, and diabetes mellitus, which are all cardiovascular diseases, are very common in the modern population. However, with the development of the times, it is gradually recognized that a diet rich in plant foods can prevent or improve the above-mentioned metabolic diseases by providing various micro and bulk nutrients, namely, minerals, vitamins, dietary fiber, and various phenolic compounds.

Owing to excellent antioxidant activity, extracts from pomegranate peels can inhibit the oxidation of low-density lipoproteins, thereby delaying the progression of atherosclerosis and significantly reducing the level of arterial foam cells (81–83). Pomegranate peel powder is rich in dietary fiber, which can also treat hypercholesterolemia and atherosclerosis. The addition of 5, 10, and 15/100 g of peel powder to the diet for 4 weeks significantly lowered the contents of serum total cholesterol, triglycerides, low-density lipoprotein, and lipid peroxidation in hypercholesterolemic rats (84). In 2017, Stockton et al. (85) conducted a clinical trial in healthy volunteers. One group took one capsule per day of the placebo. The other group took pomegranate extract containing 210 mg of punicalagin, 328 mg of pomegranate polyphenols, such as flavonoids and ellagic acid, and 0–37 mg of anthocyanins. Key vital signs were detected during the study period (4 weeks), showing that pomegranate can reduce diastolic blood pressure. Thus pomegranate played a key role in preventing some cardiovascular risk factors (such as hypertension) (86). However, the evidence of cardiovascular protection after PPE depletion remains insufficient and further studies are still needed owing to the high heterogeneity between trials and inter-individual variability.

### Others

The above physiological functions of pomegranate peels are shown in Table 1. In addition to them, punicalin, punicalagin, gallic acid, and ellagic acid, active substances in pomegranate peels, may play a vital role in antiviral, modulating respiratory infections and influenza. The antiviral properties are mainly due to the fact that the polyphenol extracts inhibit the RNA replication of influenza viruses. Viral RNA replication was maximally blocked when punicalagin was applied at concentrations up to 40 mg/mL (87). Improvement in epithelialization, fracture strength, and shrinkage of cut wounds were observed after treatment with PPE (69). In another study, oral administration of 100 mg/kg pomegranate peel aqueous extracts to Wistar rats and topical application of a hydrophilic gel formulation of PPE significantly improved all trauma models (88). Furthermore, animals fed diets rich in PPE showed signals of neuroprotection related to the biological activity of ellagic acid and its derived metabolite urolithin, suggesting that the active substances in pomegranate peel may prevent or intervene in Alzheimer’s disease (89, 90).

**TABLE 1:** Summary of physiological functions of pomegranate peels.

Physiological functions	Bioactive compounds	Evidences of effects
Antioxidant	Ellagitannins	• Urolithin as an antioxidant molecule, may reduce cisplatin-induced renal oxidative damage in the mice’s kidneys *in vivo* (51). • Pomegranate peel extracts reduce the levels of TBARS, Ox-LDL, lipid peroxidation, and oxidative biomarkers associated with cardiovascular risk in healthy volunteers (52–54).
Anti-inflammatory	Urolithins	• PPE exerted anti-inflammatory effects by significantly reducing inflammatory markers (MDA, IL-6, and hs-CRP) in obese volunteers (61). • In 2010, an *in vivo* study on intestinal inflammation found that Uro-A is a potent anti-inflammatory molecule (62).
Anticancer	Punicalagin, ellagic acid, ellagitannins	• Punicalagin and ellagic acid induce apoptosis in prostate cancer cells through some basic pathways, such as the introduction of cytochrome *c* in the cell cytoplasm, upregulation of Bax, and downregulation of Bcl-2 (64). • Urolithin B, a metabolite of ellagitannin, can inhibit testosterone-induced proliferation of MCF-7 cells associated with breast cancer (69). • Pomegranate peel extracts can inhibit the cell proliferation and the expression of angiogenic markers, and activate pro-survival signal pathways (70, 71).
Anti-bacterial	Punicalagin, ellagitannins, ellagic acid, gallic acid	• Phenolic compounds can make membrane proteins precipitated and inhibit the activity of enzymes, thus leading to bacterial death (74–77). • Punicalagin, ellagitannin, ellagic acid, and gallic acid can treat the *Staphylococcus aureus* and hemorrhagic *Escherichia coli* (78–80).
Cardiovascular protection	Polyphenols, dietary fiber	• The polyphenols and dietary fiber in pomegranate peels prevent cardiovascular disease by lowering serum total cholesterol, triglycerides, LDL, and lipid peroxidation levels (84).
Others	Punicalin, punicalagin, gallic acid, ellagic acid, urolithins	• The antiviral properties are mainly due to the polyphenol extracts inhibits the RNA replication of influenza viruses (87). • Ellagic acid and its derived metabolite urolithin may prevent or intervene in Alzheimer’s disease (89, 90).

## Conclusion

Despite the limitations remain, PPEs demonstrate some potentially beneficial effects by improving specific disease biomarkers. As a valuable by-product, pomegranate peel contains bioactive substances, especially phenolic compounds such as tannins, flavonoids, and polyphenols, which can exhibit superior biological activity. PPE showed potential antioxidant activity by reducing the levels of oxidative biomarkers such as TBARS, Ox-LDL, and lipid peroxidation in healthy volunteers. Punicalagin and ellagic acid in pomegranate peel have chemopreventive effects against prostate cancer, breast cancer, colon cancer, partly associated with the ellagic-acid derived metabolite urolithin. In addition, bioactive substances in pomegranate peels play key roles in anti-inflammatory, antibacterial, improvement of cardiovascular diseases, anti-infection and healing, playing a modulating and interventional role.

However, whether the observed changes are clinically relevant warrants additional studies in the general population through well-designed randomized controlled trials. Besides, the actual effects of clinical application require more studies in the general population by well-designed and randomized controlled trials. Similarly, clinical evidences for the benefits of ingesting pomegranate peel derivatives on anti-inflammatory and anti-cancer processes are still limited. In addition, the relevant molecular mechanisms are not yet clear. Therefore, the impact and possible toxicological effects of these natural extracts on human health should still be addressed through preclinical and clinical trials. Based on this, pomegranate peels can be effectively utilized as a rich potential by-product resource. They can be applied to animal feed to improve feed efficiency. Moreover, the bioactive substances in pomegranate peels can be used as natural food ingredients to prepare innovative food products. What’s more, the various physiological functions of pomegranate peel active substances can provide auxiliary strategies for the treatment of related human diseases.

## Author Contributions

YM and JM conceptualized the topic, analyzed the literature, and wrote the manuscript. WG and LZ researched the background literature. JML and JZ provided the extensive academic guidance and critically revised the manuscript. All authors approved the submitted version, made the work accurate, and agreed to take responsibility for the work.

## Conflict of Interest

The authors declare that the research was conducted in the absence of any commercial or financial relationships that could be construed as a potential conflict of interest.

## Publisher’s Note

All claims expressed in this article are solely those of the authors and do not necessarily represent those of their affiliated organizations, or those of the publisher, the editors and the reviewers. Any product that may be evaluated in this article, or claim that may be made by its manufacturer, is not guaranteed or endorsed by the publisher.

## Abbreviations

Uro-A: urolithin A
Uro-B: urolithin B
TBARS: thiobarbituric acid reactive substances
Ox-LDL: oxidized low density lipoprotein
MDA: malondialdehyde
IL-6: interleukin-6
IL-1β: Interleukin-1 beta
hs-CRP: hypersensitive-C reactive protein
LCAI: Lichtiger colitis activity index
Bcl-2: B cell lymphoma/leukemia-2
PSA: prostate specific antigen
8-OHdG: 8-hydroxy-2′ -deoxyguanosine
IGF-1: insulin-like growth factor-1
IGFBP-3: insulin-like growth factor-binding protein 3
MCF-7: Michigan Cancer Foundation-7
IGFs: insulin-like growth factors
IGFBP: insulin-like growth factor binding protein
JNK: c-Jun N-terminal kinase
PKB/Akt: protein kinase B
MTOR: mammalian target of rapamycin.
